# Studying the Performance of SnS-Based Zn (0%, 2% and 4%)-Doped Methanol Sensors Under the Optimal Temperature of 240 °C

**DOI:** 10.3390/mi17060707

**Published:** 2026-06-09

**Authors:** Yaotong Ma, Xiaofeng Yuan, Fanting Kong

**Affiliations:** School of Electronics and Communication Engineering, Lanzhou University of Arts and Sciences, Lanzhou 730000, China; 13639313380@163.com (X.Y.); kfanting@163.com (F.K.)

**Keywords:** hydrothermal method, Zn-SnS, methanol gas sensor

## Abstract

Addressing the critical limitation of high operating temperatures plaguing conventional resistive gas sensors, this work reports the synthesis of Zn-doped SnS gas-sensing materials with doping concentrations of 0%, 2%, and 4% via a one-step hydrothermal route—an approach that enables precise regulation of dopant distribution and material microstructure. Systematic gas-sensing tests demonstrate that all as-prepared sensors exhibit remarkable responsiveness to methanol at a reduced optimal operating temperature of 240 °C, with the response values increasing significantly with Zn doping content: 22.1% for pristine SnS, 48.9% for 2% Zn-doped SnS, and 65.2% for 4% Zn-doped SnS when exposed to 50 ppm methanol. Beyond enhanced response, the Zn-doped SnS sensors maintain excellent methanol selectivity against interfering gases (e.g., ethanol, formaldehyde, acetone) and achieve a low detection limit of 5 ppm, which meets the practical requirements for trace methanol monitoring. The superior performance of 4% Zn-doped SnS—exhibiting a 195% response enhancement compared to pristine SnS—originates from the synergistic effects of Zn-induced defect engineering and improved charge carrier mobility, as supported by structural and electrical characterizations. This study not only provides a facile strategy for developing low-temperature-operating methanol sensors but also highlights the potential of Zn-doped SnS as a promising candidate for high-performance gas-sensing applications in environmental monitoring, industrial safety, and biomedical detection.

## 1. Introduction

Methanol is a typical chemical raw material and a high-quality clean fuel. It is a commonly used raw material for pesticides, medicines, analytical reagents, synthetic fibers, cleaning and degreasing agents, etc. Its molecular formula is CH_3_OH and it belongs to volatile organic compounds (VOCs). However, methanol is highly toxic, flammable and explosive. It can mix with alcohol in any proportion and is difficult to distinguish. The industrial alcohol (containing methanol) mixed in adulterated alcohol is harmful to the human central nervous system and circulatory system. More than 10 mL of methanol may lead to permanent blindness and fatal poisoning [1,2]. Therefore, precise monitoring and detection of methanol is very important. Currently, methods used for methanol detection include gas chromatography, ultraviolet/visible or infrared spectroscopy, and gas sensor methods. Among them, gas chromatography and spectroscopic analysis methods require expensive instruments that are difficult to carry, making it impossible to conduct timely and on-site detection, and they thus are difficult to be widely applied. In contrast, methanol detection based on gas sensors is widely used due to the significant advantages of gas sensors, such as high response, simple operation, low cost, and small size. So far, the reported methanol sensors are mainly based on metal oxide semiconductors [3]. For instance, ZnO micro-rod prepared by the hydrothermal reaction process can respond to 100 ppm methanol gas at a working temperature of 300 °C with a response of 4.41 × 10^4^% [4]. The gas sensors based on Ce-doped In_2_O_3_ porous nanospheres synthesized by the hydrothermal method have a response of 35.2 to 100 ppm methanol gas at the operating temperature of 320 °C [5]. Peilun Qiu et al. [6] fabricated a SnS/TiO_2_ memristor-type methanol gas sensor that can respond to 1 ppm methanol at room temperature with a response of 85.2. J. Guo et al. [7] demonstrated SnO_2_ porous nanosheets, which have excellent sensing performance for 50 ppm methanol gas at 300 °C. Most methanol sensors based on metal oxide semiconductors need high temperatures to work (usually 300~400 °C), but there are a series of problems such as increased power consumption and accelerated device aging. Moreover, the high detection limit is not conducive to low-concentration detection. Therefore, it is necessary to find a methanol gas-sensitive sensing material that works at a modest operating temperature, has high sensitivity, good selectivity, and a fast response time to low-concentration methanol gas.

In recent years, two-dimensional (2D) and 3D sulfides have been favored due to their excellent electrical, optical, and mechanical properties, as well as gas-sensing performance, by researchers. SnS is a new type of 2D material with a direct band gap of 1.0 eV and an indirect band gap of 1.3 eV, which can be applied in fields such as photovoltaic cells, photodetectors and sensors materials [8,9,10]. It is important to note that the gas sensors constructed using SnS nanosheets recently have been proven to respond to gaseous VOCs and NO_2_ at room temperature [11]. Two-dimensional sulfide gas sensors can display a rapid response because of the rapid charge transfer between gas molecules and the substrate. Furthermore, the electronegativity of S atoms is smaller than O, making them more easily absorb O in the air. Therefore, S-based gas sensors usually work at much lower temperatures than oxide semiconductor sensors [12,13].

Currently, effective methods for improving the gas-sensing performance of semiconductors include noble metal modification, constructing heterojunction structure and doping. In particular, doping impurity atoms has been proven as a simple and effective method for enhancing gas sensitivity, which can disrupt the lattice structure, cause defects in the gas-sensitive material and provide more adsorption sites. Additionally, constructing a hierarchical structure also facilitates reduced aggregation or stacking of 2D nanosheets. Loose-layered structures usually have a larger active surface area that can be used for gas adsorption and diffusion, thus facilitating the achievement of high gas sensitivity response. This paper successfully synthesized pure Zn with different doping ratios of SnS gas-sensing materials using a simple one-step hydrothermal method and conducted further experimental studies. The sensing responses of the synthesized pure SnS and substituted Zn to methanol vapor exposure confirmed that doping Zn significantly enhanced the gas-sensing response of SnS to methanol and the response value of 4% Zn-doped SnS (abbreviated label: ZS) increased more than 2.9 times compared with pure SnS.

## 2. Materials and Methods

SnS and Zn-SnS were synthesized using a one-step hydrothermal method. First, 1 mmol (0.226 g) of SnCl_2_·2H_2_O and 0, 0.272, and 0.544 g of ZnCl_2_ (0, 2%, and 4% molar mass), and 2 mmol (0.114 g) of thiourea (CH_4_N_2_S) were added into 30 mL of ethylene glycol (CH_2_(OH)_2_CH_2_). Ultrasound and magnetic stirring were alternately performed to ensure complete dissolution of the mixture. Then, 1 mmol (0.210 g) of monohydrate citric acid (C_6_H_8_O_7_·H_2_O) was added to the precursor solution and the mixture was further subjected to ultrasound and magnetic stirring for 3 h. The mixture was then transferred to a 80 mL polytetrafluoroethylene inner container hydrothermal reactor and heated at 190 °C for 24 h. After cooling to room temperature naturally, the samples were washed with alternating amounts of deionized water and anhydrous ethanol, and then dried at 70 °C for 24 h to obtain pure samples of SnS (0% ZS), (2% ZS), and (4% ZS) powders.

**Scheme 1:**
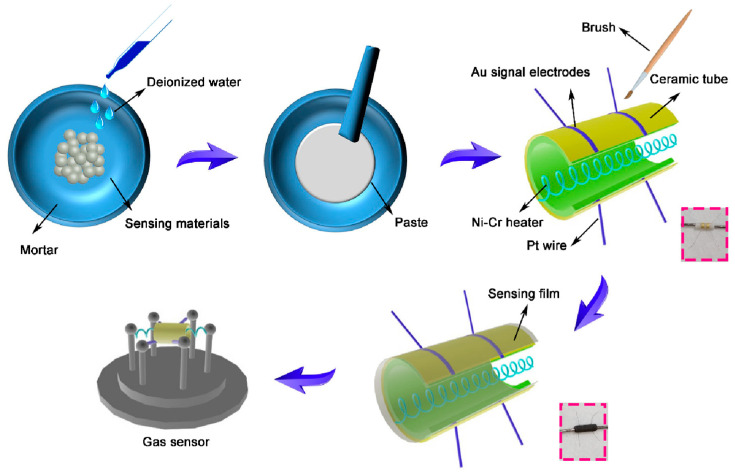
Schematic diagram of the fabrication procedure of the gas sensor.

The crystal structure of the samples was tested by XRD (D/Max-2400, Japan) with Cu Kα1 (λ = 0.15406 nm) as the diffraction source and the test results were analyzed. The micro-structure and morphology of the samples were characterized by SEM (TESCAN MIRA3, Czech Republic). The gas-sensing properties of the samples were evaluated by WS-30B gas-sensing analysis and testing system (Zhengzhou Weisheng Electronic Technology Co., Ltd., Zhengzhou, China). The sensor response to the target gas was calculated using the formula:

*S* = ∣Δ*R*∣/*R*_a_ × 100% = ∣*R*_a_ − *R*_g_∣/*R*_a_ × 100%

where *R*_a_ is the resistance of the sensor in air and *R*_g_ is the resistance of sensor in the target gas. The fabrication process of the gas sensor (Scheme 1) is similar to a previous work [14,15].

## 3. Results

### 3.1. XRD Analysis

**Figure 1:**
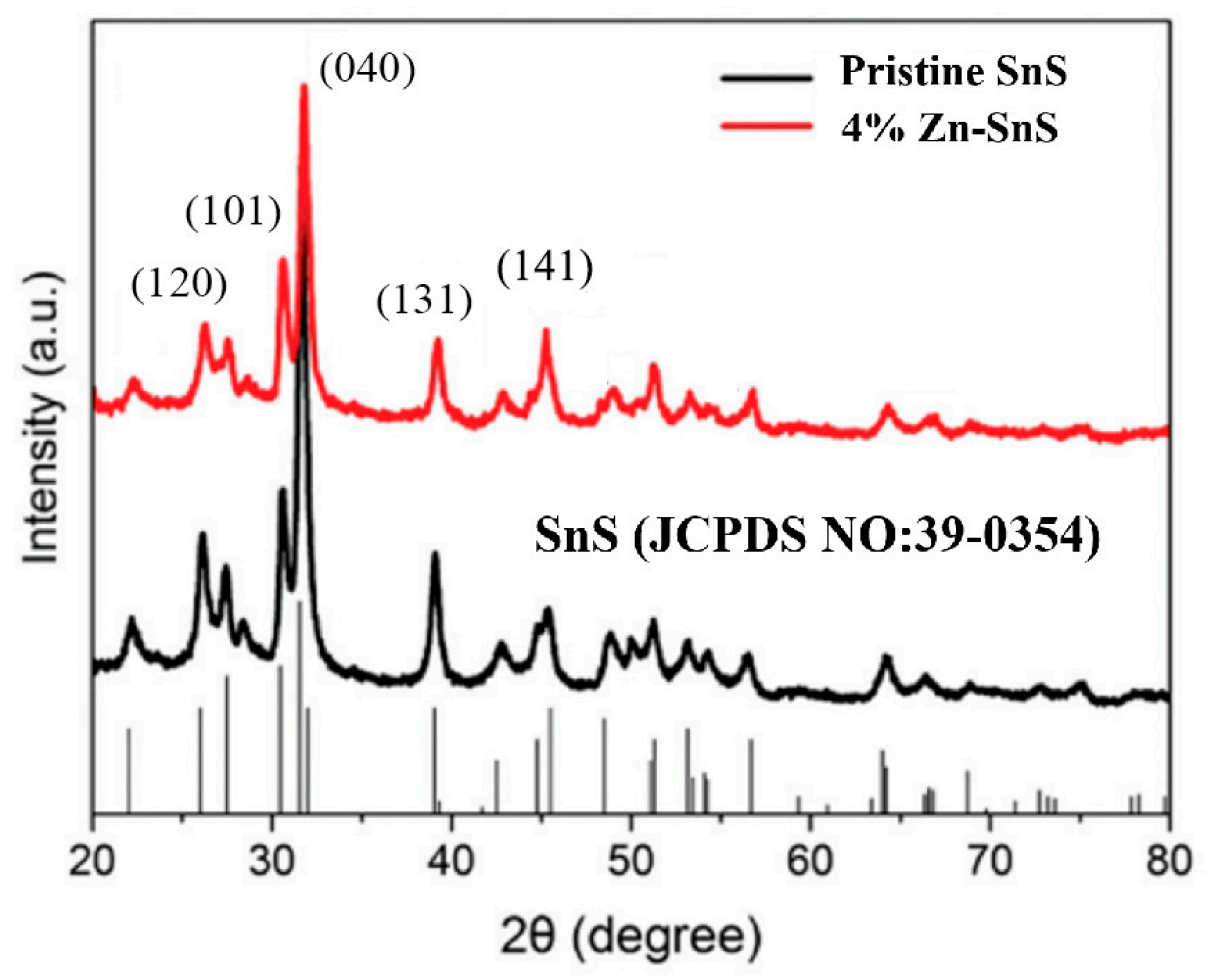
XRD patterns of SnS and 4% Zn-SnS samples.

The chemical composition of pristine SnS and 4% Zn-SnS samples was analyzed using an X-ray diffractometer. The results are shown in Figure 1. As can be seen from Figure 1, the XRD spectrum exhibited the strongest characteristic peak at the diffraction angle 2θ of 31.8°, and secondary strong peaks at 26.3°, 30.4°, 38.9°, and 45.1°. By comparing with the standard card (JCPDS No. 39-0354), they corresponded to the (040) crystal plane and (120), (101), (131), and (141) crystal planes of the orthorhombic crystal of SnS. Additionally, it was found that after doping with 4% Zn, the main diffraction peaks slightly shifted towards larger angles, confirming that the smaller ionic radius of Zn^2+^ (*R*_Zn_ = 74 pm) has successfully replaced Sn^2+^ (*R*_Sn_ = 112 pm) in the SnS lattice [16]. For the 2% Zn-SnS samples, XRD test results were almost identical to those of the pure SnS samples. This might be due to the insufficient Zn doping amount. No other impurity peaks appeared in the XRD spectrum, indicating that high-purity SnS and 4% ZS samples were successfully prepared.

### 3.2. SEM Analysis

**Figure 2:**
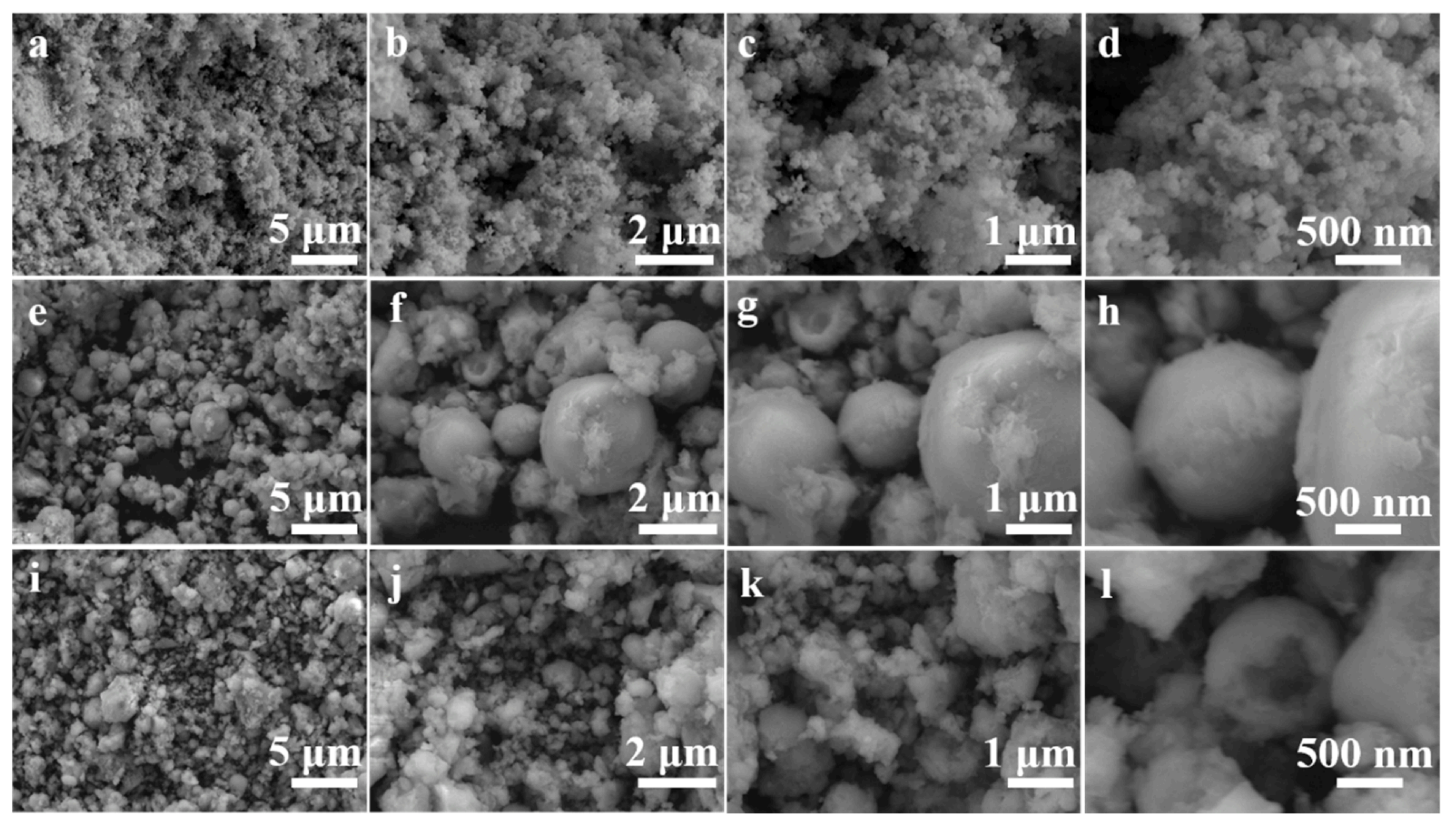
SEM images of the samples: (**a**–**d**) SnS; (**e**–**h**); 2% Zn-SnS; and (**i**–**l**) 4% Zn-SnS.

In order to observe the microstructure and morphology of the prepared products, scanning electron microscopy (SEM) technology was employed on SnS, 2% ZS and 4% ZS samples, as shown in Figure 2. It can be see that the pure sample SnS (Figure 2a–d), the 2% ZS (Figure 2e–h) and 4% ZS (Figure 2i–l) all present a three-dimensional (3D) spherical hierarchical structure composed of self-assembled ultra-small nanocrystals. The nanocrystals support each other and form a loose hollow spherical structure. The morphology of Zn-doped SnS is quite similar to that of the pure sample SnS, indicating that Zn does not change the spherical structure of SnS. However, Zn atoms were actually doped into the lattice of SnS, which coincides with the analytical results of the right-skewed XRD peak after doping. Notably, hollow spherical products with larger particle sizes appeared after doping. This change is because Zn is more chemically active than Sn and is more prone to agglomeration during the hydrothermal process. No other morphologies were detected, indicating that the yield of 3D microstructures is very high under the current hydrothermal conditions. Meanwhile, it is possible that the multi-level hierarchical structure composed of nanocrystals has high stability.

### 3.3. Gas-Sensing Performance

**Figure 3:**
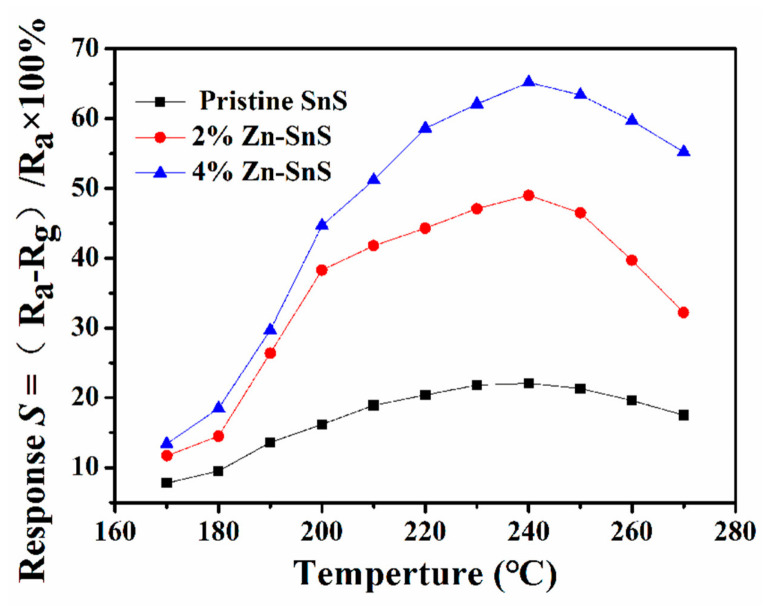
Responses of SnS, 2% Zn-SnS, and 4% Zn-SnS sensors to 50 ppm methanol at different temperatures.

At present, the gas sensors for detecting methanol are mainly based on metal oxide semiconductors and their optimal operating temperature from 200 to 400 °C [17]. On the basis of previous work [15], SnS-, 2% ZS- and 4% ZS-based sensors were first selected to test the response of 50 ppm methanol at different temperatures. The results are shown in Figure 3. The operating temperature affects the chemical adsorption state of oxygen ions on the material surface, as well as the surface oxidation/reduction reaction and the performance of sensing.

From Figure 3, we can see that the optimal operating temperature for all three samples is 240 °C. Moreover, Zn-doped SnS can significantly improve the response to methanol gas and the higher doping ratio, the larger the response value (S) among the three sensors at the same temperature, and 4% ZS has the greatest response to methanol. The responses of the three sensors to 50 ppm methanol all showed a trend of “increase—maximum—decrease” with the increase in temperature. The sample of 2% ZS came second while the pure sample of SnS had the smallest response, with responses of only 7.8% at 170 °C and 22.1% at 240 °C. This phenomenon can be attributed to the fact that pure samples of sulfides can provide limited oxygen vacancies compared with oxides and sulfides, which usually work at lower temperatures than oxides. Many research teams have developed metal-doped sulfide sensors that can detect gases at room temperature [18]. Without exception, the responses of the three sensors to methanol decrease when the temperature exceeds 240 °C; rapid response is limited because gas molecules gain sufficient energy to quickly escape from the material surface, affecting the conductivity of the sensor and leading to a decrease in response.

**Figure 4:**
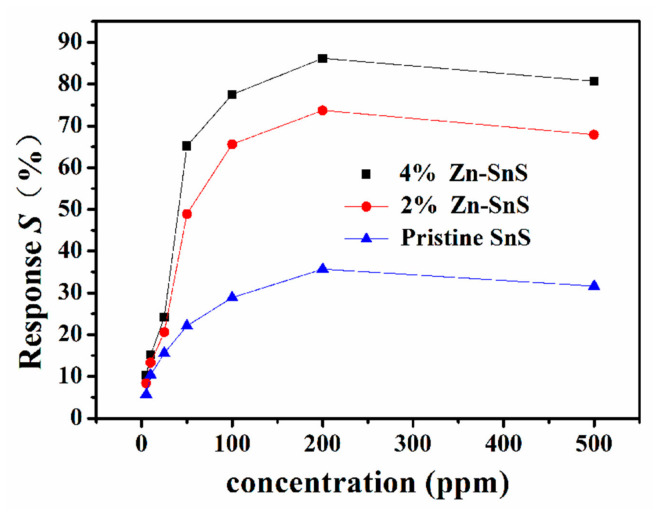
Responses of SnS, 2% Zn-SnS, and 4% Zn-SnS sensors to methanol at different concentrations (5–500 ppm) at 240 °C.

Figure 4 shows the response of pure SnS-, 2% ZS- and 4% ZS-based sensors to different concentrations of methanol at the optimal operating temperature of 240 °C, respectively. It can be seen that the response of 4% ZS is the largest and the response of pure SnS is the smallest among the three sensors under the same concentration. When the concentration of methanol increased from 5 ppm to 200 ppm, the responses of the three sensors increased by 5.7–35.7% (pure SnS), 8.4–73.7% (2% ZS), and 10.3–86.2% (4% ZS), respectively. The responses of all sensors decrease when the concentration of methanol exceeds 200 ppm, indicating that 200 ppm is adsorption limit for the three materials. In this case, the adsorption is supersaturated and the methanol vapor filled in the gas chamber cannot be fully adsorbed onto the material surface, either via physical adsorption or chemical adsorption. It should be noted that the three materials respond to methanol with the minimum concentration of 5 ppm at 240 °C, showing that sensors have a detection limit. Among the tested materials, the 4% ZS is more suitable for manufacturing low-concentration methanol sensors. Some scholars have also predicted that ZS can detect methanol at the ppb concentration level at room temperature through theoretical calculations [16].

**Figure 5:**
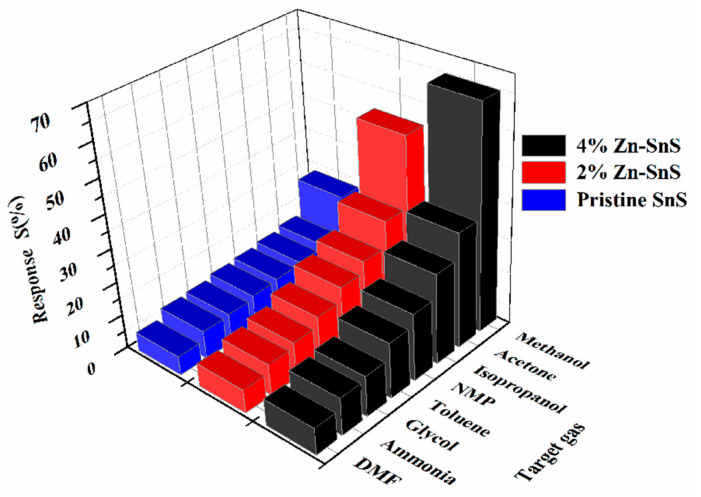
Selectivity of SnS, 2% Zn-SnS, and 4% Zn-SnS sensors toward different VOC target gases (50 ppm) at 240 °C.

The selectivity of SnS-, 2% Zn-SnS- and 4% Zn-SnS-based sensors for several different 50 ppm VOC target gases, including methanol, acetone, isopropanol, NMP (N-methylpyrrolidone), toluene, ethylene glycol, ammonia and DMF (dimethylformamide), were tested at 240 °C. As demonstrated in Figure 5, the responses of the three different sensors to methanol are 4.2~9.5 times higher than other VOCs, confirming that they all have excellent selectivity for methanol. Yu xiang Qin et al. [16] once used the first principles of density functional theory (DFT) to calculate the adsorption energy and adsorption distance of pure SnS and Zn-SnS to methanol and other VOC gases. It was found that the adsorption energy of SnS for methanol was 0.35 eV and the adsorption distance was 2.676 Å, while for ZS these were 0.62 eV and 2.427 Å. This theoretical calculation result validates that the adsorption energies of Zn-SnS is higher and that the adsorption distance is shorter to methanol than to other VOC gases. This can provide a reasonable explanation for the excellent selectivity of SnS and ZS for methanol from both experimental and theoretical perspectives.

**Figure 6:**
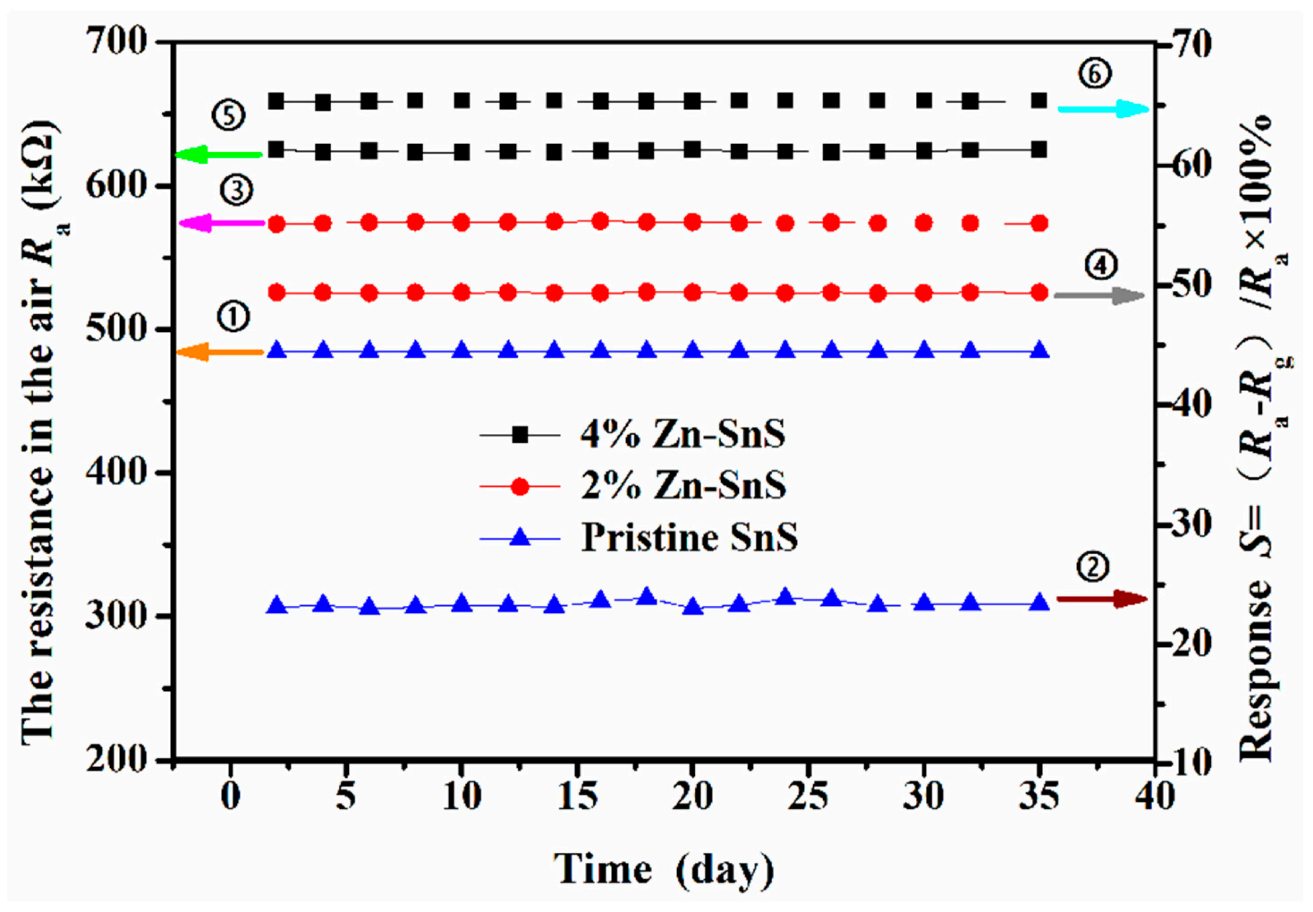
Long-term stability of SnS, 2% Zn-SnS, and 4% Zn-SnS sensors over 35 days.

In order to evaluate the long-term stability and repeatability of SnS-, 2% ZS- and 4% ZS-based sensors, uninterrupted tests were conducted on the resistance (*R*_a_) of the sensors in air at room temperature and their response (*S*) in 50 ppm methanol atmosphere at 240 °C within 35 days (5 weeks), as depicted in Figure 6. Across the 5 weeks, the *R*_a_ of the three sensors (① SnS, ③ 2% Zn-SnS, ⑤ 4% Zn-SnS) was relatively stable. The variation range of the response *S* (② SnS, ④ 2% Zn-SnS, ⑥ 4% Zn-SnS) was also very small. It was indicated that SnS-, 2% Zn-SnS- and 4% Zn-SnS-based sensors all have relatively excellent stability.

The gas-sensing results demonstrate that Zn doping effectively enhances the methanol response of SnS. This improvement is primarily attributed to the smaller ionic radius of Zn^2+^ compared with Sn^2+^, resulting in a shorter Zn-S bond relative to the Sn-S bond. The stronger Zn-S bonding modifies the electronic environment, making the formation of Sn vacancies more difficult. Consequently, the concentration of Sn vacancies decreases, leading to a reduction in effective hole carrier density [16]. As reflected in Figure 6, the baseline resistance of SnS increased with Zn doping, with measured values of 484.37 kΩ for SnS, 573.92 kΩ for 2% Zn-SnS, and 625.16 kΩ for 4% Zn-SnS. The substitution of Zn^2+^ for Sn^2+^ also slightly deteriorates the crystal quality, which in turn creates more adsorption sites, thereby enhancing gas-sensing performance. In addition, Zn doping increases the adsorption energy and decreases the adsorption distance for methanol molecules, further contributing to the improved sensitivity of Zn-SnS-based sensors.

## 4. Conclusions

SnS samples doped with 0%, 2%, and 4% Zn were successfully synthesized using a hydrothermal method and subsequently fabricated into gas sensors. X-ray diffraction (XRD) analysis confirmed the absence of impurity phases, indicating high sample purity. Scanning electron microscopy (SEM) revealed that all samples possessed well-defined morphologies with uniform microstructures. Gas-sensing measurements demonstrated that the 0%, 2%, and 4% Zn-SnS-based sensors exhibited strong responses to methanol at an operating temperature of 240 °C. Among them, the 4% Zn-SnS sensor achieved the highest response value of 65.2% to 50 ppm methanol, along with excellent selectivity for methanol detection. In addition, the Zn-SnS sensors showed relatively low detection limits, suggesting their strong potential for the development of efficient and reliable methanol gas sensors.

## Data Availability

The original contributions presented in this study are included in the article. Further inquiries can be directed to the corresponding author.
